# Vertical Transmission of *Wolbachia* Is Associated With Host Vitellogenin in *Laodelphax striatellus*

**DOI:** 10.3389/fmicb.2018.02016

**Published:** 2018-08-28

**Authors:** Yan Guo, Ary A. Hoffmann, Xiao-Qin Xu, Pei-Wen Mo, Hai-Jian Huang, Jun-Tao Gong, Jia-Fei Ju, Xiao-Yue Hong

**Affiliations:** ^1^Department of Entomology, Nanjing Agricultural University, Nanjing, China; ^2^School of BioSciences, Bio21 Institute, The University of Melbourne, Parkville, VIC, Australia

**Keywords:** *Wolbachia*, vertical transmission, vitellogenin, tropharium, nutritive cord, oocyte

## Abstract

*Wolbachia* in host germ lines are essential for their vertical transmission to the next generation. It is unclear how the regulation of host oocyte development influences *Wolbachia* location and the mechanistic basis of transmission. Here, we investigated whether vitellogenin influences *Wolbachia* transmission in *Laodelphax striatellus*. *Wolbachia* increased in density and spread from the anterior tropharium to developing oocytes as ovaries developed. Microscopic observations indicated that *Wolbachia* invaded ovarioles from the tropharium of its anterior side rather than the pedicel side. *Wolbachia* utilized the host Vg transovarial transportation system to enter the ovaries and were transmitted from the tropharium into the developing oocytes through nutritive cords. These observations were supported by knocking down the Vg transcript, in which low *Wolbachia* titers were detected in ovaries and fewer *Wolbachia* were transmitted into oocytes. Our findings establish a link between the Vg-related mode of transovarial transmission and efficient maternal transmission of *Wolbachia*.

## Introduction

*Wolbachia* bacteria that occur intracellularly in reproductive tissues of a wide range of arthropods (O’Neill et al., 1992; Rousset et al., 1992; Werren et al., 1995) are vertically transmitted from parents to offspring in a transovarial manner. The transmission process controls the ability of *Wolbachia* to persist and disperse (Schmidt et al., 2017), particularly when coupled with effects on their hosts that enhance *Wolbachia* transmission, including induction of cytoplasmic incompatibility between individuals (Breeuwer and Werren, 1990; Rousset and Raymond, 1991; Turelli and Hoffmann, 1995), male-killing(Hurst et al., 1999), parthenogenesis induction (Stouthamer et al., 1993), feminization (Rousset et al., 1992), and positive host fitness effects (Weeks et al., 2007). To be vertically transmitted, *Wolbachia* need to invade host reproductive tissues to ensure their presence in the germ line.

*Wolbachia* symbionts are localized in diverse cells and tissues (Dobson et al., 1999), exhibiting strong tropism (Toomey et al., 2013). Although *Wolbachia* have been detected in host somatic tissues, they are primarily found in the host germ line (Dobson et al., 1999; Clark et al., 2002; Ijichi et al., 2002). In male arthropods, *Wolbachia* are present within developing spermatocytes (Bressac and Rousset, 1993; Clark et al., 2002; Ijichi et al., 2002), but they are not transmitted through sperm. In female arthropods, *Wolbachia* are transmitted to eggs, where they persist through embryogenesis and eventually become incorporated in the precursors of germ line stem cells (Kose and Karr, 1995; Ferree et al., 2005). *Wolbachia* accumulate in the germ line stem cells in long-term maternally infected *Drosophila mauritiana* (Fast et al., 2011). In *D. melanogaster* infected with *Wolbachia* through microinjection, *Wolbachia* enter the germ line through somatic stem cells (Frydman et al., 2006). Both host- and bacterial- derived factors appear to influence *Wolbachia*’s tropism during oogenesis (Toomey et al., 2013; Hughes et al., 2014).

Besides *Wolbachia*, other microbes including viruses, microsporidia, and fungi can be maternally transmitted (Douglas, 1989). *Spiroplasma* in *D. melanogaster* achieve vertical transmission by cooption of the yolk uptake machinery (Herren et al., 2013). In planthoppers, these microbes have evolved strategies to enter the reproductive tissues of the host involving vitellogenin (Vg). Vg, a precursor of egg yolk protein, is associated with egg maturation, embryonic growth and oviposition capacity (Dong et al., 2007; Kawakami et al., 2009; Guo et al., 2012). After its synthesis by fat body, Vg is secreted into hemolymph, absorbed into ovaries through wide channels formed between epithelium cells, then transferred toward the oocyte surface and into the oocyte via receptor-mediated endocytosis (Davey, 1993; Zimowska et al., 1994). There is a transovarial transportation system for absorbing Vg in the oviparous insects. Yeast-like symbionts (YLS) in brown planthoppers are transovarially transmitted by being wrapped in vitellogenin outside the ovary (Cheng and Hou, 2005). In the small brown planthopper *Laodelphax striatellus* (Hemiptera: Delphacidae), *rice stripe virus* (RSV) can be transmitted to oocytes only when the Vg receptor is present (Huo et al., 2014). Maternal transmission of these microbes in planthoppers is therefore potentially related to Vg, and this protein may also be associated with the efficient maternal transmission of *Wolbachia*.

**FIGURE 1:**
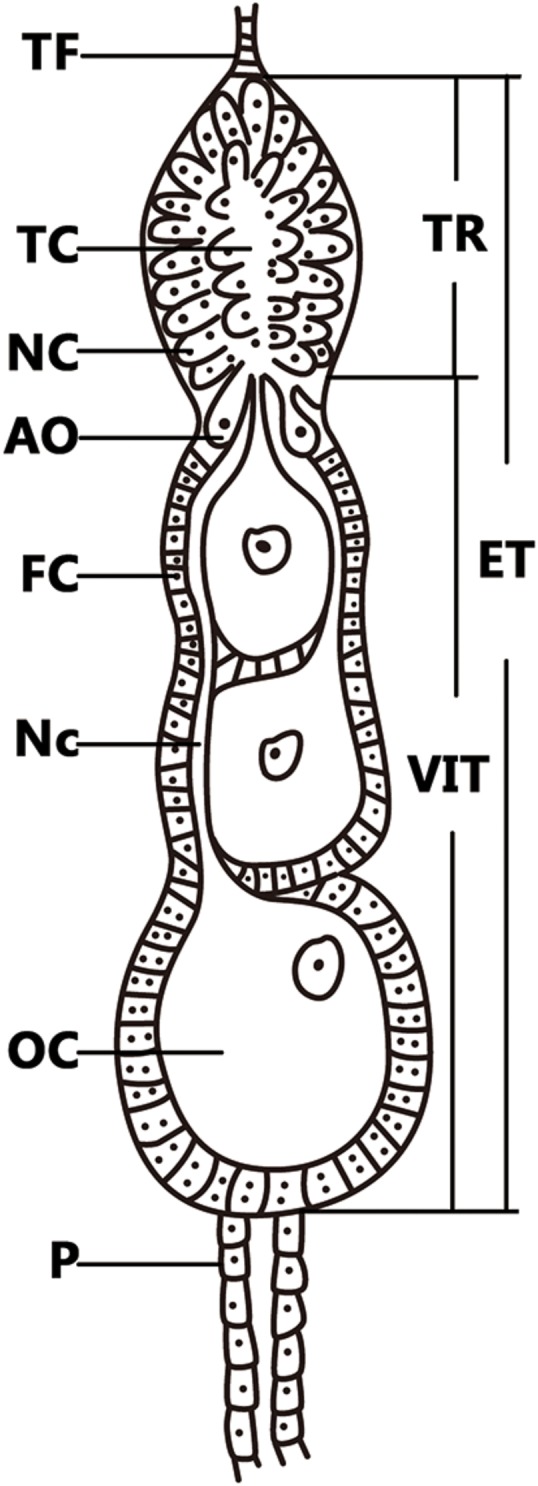
Telotrophic meroistic ovaries of *L. striatellus.* The *L. striatellus* ovariole is composed of a terminal filament (TF) at the tip, followed by the egg tube (ET). The tropharium (TR) is at the tip of the egg tube, which is composed of nurse cells (NC) and a trophic core (TC); just below the tropharium is the vitellarium (VIT), in which the oocyte (OC) is completed and there is a pedicel (P) at the bottom. The posterior region of the tropharium contains 6–10 arrested oocytes (AO). The vitellarium houses linearly arranged developing oocytes each of which is connected to the trophic core by a broad nutritive cord (Nc). Each oocyte is surrounded by a single layer of follicular cells (FC) that become binucleate at the beginning of vitellogenesis.

The small brown planthopper, *L. striatellus* is an important pest in rice fields and is 100% infected with *Wolbachia* strain *w*Stri in China (Zhang et al., 2013). *Wolbachia* transovarial transmission frequencies were determined to be 100% from mother to offspring (Noda et al., 2001; Zhang et al., 2013), which indicates that *Wolbachia* are capable of efficient transmission via *L. striatellus* ovaries. *L. striatellus* ovarioles are of the telotrophic meroistic type, which consist of a terminal filament, tropharium, and vitellarium (**Figure 1**). In anterior tropharium, a cluster of nurse cells are radially arranged around and connected to the central trophic core; previtellogenesis are arranged on the base of the tropharium (Szklarzewicz et al., 2007). The vitellarium house 3-4 linearly arranged developing oocytes which absorb nutrients from nurse cells through nutritive cords that connect nurse cells and oocytes (Szklarzewicz et al., 2009; Szklarzewicz et al., 2013). Based on oogenesis development, ovaries can be divided into four stages: previtellogenic, early vitellogenic, vitellogenic, and postvitellogenic stages (Dong et al., 2011). *Wolbachia* titers increased in females after *L. striatellus* adult emergence as ovaries developed (Noda et al., 2001). However, the mechanism by which *Wolbachia* spreads into host ovaries is unknown, and host proteins that are involved in *Wolbachia* overcoming barriers to transovarial transmission have not been directly characterized.

Here we describe how *Wolbachia* is transmitted to *L. striatellus* oocytes and where the bacteria are located in the ovarioles. Our results provide a model for *Wolbachia* transovarial transmission into host telotrophic meroistic-type ovaries, and they indicate that the host Vg transovarial transportation system plays a role in this process.

## Materials and Methods

### Insects

The *Wolbachia*-infected *L. striatellus* population was originally collected from rice fields in Nanjing, Jiangsu, China, and maintained in an incubator at 26 ± 1°C under a 16: 8 h light: dark photoperiod and 60% relative humidity on rice variety TN_1_ rice plants. Before experiments, the genotype of *Wolbachia* strain *w*Stri was determined by cloning and sequencing five multilocus sequence typing genes and *wsp*, as previously described (Zhang et al., 2013). To ensure a similar nuclear background, all *Wolbachia*-infected insects were from offspring of one infected pair.

### Ovarian Development

*Laodelphax striatellus* ovarian development was divided into previtellogenic, early vitellogenic, vitellogenic, and postvitellogenic different stages, as described in *Nilaparvata lugens* (Dong et al., 2011), which is related to *L. striatellus*. Nearly no Vg is transported into the previtellogenic ovaries of adults within 12 h after eclosion. Most ovarioles were in early vitellogenesis within 1–2 days after eclosion when Vg began to be transported into oocytes from hemolymph; they were transparent and there was little yolk protein deposition in the oocytes. Vitellogenic ovarioles contain one white and opaque oocyte, with nearly half of the oocytes filled with yolk protein and oocytes gradually increasing in size. Most ovarioles in the postvitellogenic ovaries contained one full-grown egg, which was completely filled with yolk granules and surrounded by the vitelline membrane.

### Reverse-Transcriptase Quantitative PCR (RT-qPCR)

Nearly no egg was laid within 72 h after *L. striatellus* female eclosion. Vg expression levels in 5th instar nymphs and three stages of 1-, 2-, and 3-day-old adult female *L. striatellus* were measured by RT-qPCR. Total RNA was extracted using TRIZOL reagent (Takara) and converted to cDNAs using PrimeScript^TM^ RT reagent Kit with gDNA Eraser (Takara), according to the manufacturer’s instructions. Relative Vg mRNA levels were measured with the SYBR Primex Ex Tap^TM^ Kit (Takara Biotechnology) following the manufacturer’s instructions. RT-qPCR was performed in 20 μl reactions containing 10 μl SYBR Primex Ex Tap, 2 μl of diluted cDNA template, 0.4 μl of each primer, 0.4 μl ROX Reference Dye II, and 6.8 μl ddH_2_O, on a 7800 real-time system (Applied Biosystems) using the following procedures: denaturation at 95°C for 30 s, followed by 40 cycles of amplification (95°C for 5 s and 60°C for 34 s). The primers are listed in **Supplementary Table 1**. The dissociation curve was analyzed for both primer pairs, and all experimental samples had a single sharp peak at the amplicon’s melting temperature. The relative expression levels of the Vg gene were calculated according to the 2^-ΔΔCT^ method (Livak and Schmittgen, 2001), and the actin gene of *L. striatellus* was used as a housekeeping gene. All the RT-qPCR experiments were repeated five times. All the data were presented as relative mRNA expression (mean ± SD).

### qPCR

*Wolbachia* densities in 5th instar nymphs and three stages of 1-, 2-, and 3-day-old adult female *L. striatellus* ovaries were determined by qPCR.DNA extraction, qPCR protocols, and standard curves have been previously described (Lu et al., 2012). The primers (**Supplementary Table 1**) specifically designed for the surface protein gene *wsp* of *w*Stri were used in qPCR to measure *Wolbachia* genome copy number. The *Wolbachia wsp* copy number was normalized to the host actin gene.

### RNA Interference (RNAi)

*Laodelphax striatellus* Vg-specific nucleotide conserved sequence was cloned into the pGEM-T Easy vector (Promega). *Aequorea victoria* green fluorescent protein (GFP) was used as a control. The specific primers used to generate DNA templates are shown in **Supplementary Table 1**. dsRNA was synthesized, as previously described (Huang et al., 2015).

Second instar nymphs of infected *L. striatellus* were used in the RNAi microinjection. Each nymph was anesthetized using carbon dioxide. Approximately 250 ng of dsVg or dsGFP was microinjected into the thorax of each nymph using the FemtoJet Microinjection System (Eppendorf). Microinjected nymphs were reared under laboratory conditions. Relative silencing efficiency of the whole body was calculated by RT-qPCR. *Wolbachia* titers (ovaries and whole body) were measured by qPCR, as described above.

### Immunofluorescence Staining

Ovaries in different development stages were stained as previously described (Frydman and Spradling, 2001; Ferree et al., 2005). Ovaries from *Wolbachia*-infected adult female *L. striatellus* were dissected in phosphate buffered saline (PBS) and separated into individual ovarioles. Ovarioles were fixed in 4% formaldehyde solution plus 0.1% Triton X-100 (Sigma) for 15 min and washed twice in PBS for 5 min each; this was followed by two 20-min washes in PBS containing 0.2% Triton X-100 and 1 U/ml of fluorescein isothiocyanate (FITC)-conjugated phalloidin (Sigma), and another 20-min wash in PBS containing 0.2% Triton X-100 without phalloidin. The ovaries were then rinsed in PBS alone, after which they were ready for antibody staining.

*Wolbachia* were stained with mouse antibody Hsp-60 (1:200) (Sigma). Primary antibodies were incubated overnight at 4°C. Following six 15-min washes in PBS with 0.1% Triton-X-100, ovaries were incubated at room temperature for 1 h with tetramethylrhodamine isothiocyanate (TRITC)-coupled anti-mouse antibodies (1:200) (Sigma). After incubation, the ovarioles were washed in PBS and mounted on a glass slide with the 4′, 6-diamidino-2-phenylindole (DAPI)-containing mounting Vectashield (Vector Laboratories).

Images were generated using a Zeiss LSM-800 confocal laser scanning microscope. Care was taken to normalize the exposure times across all experiments. Taking in consideration that each *Wolbachia* cell was about 1.0 μm in diameter, *Wolbachia* titers in ovaries were scanned with Z-series stacks at 1.0-μm intervals.

## Results

### *Wolbachia* Increase Is Correlated With Vg

**FIGURE 2:**
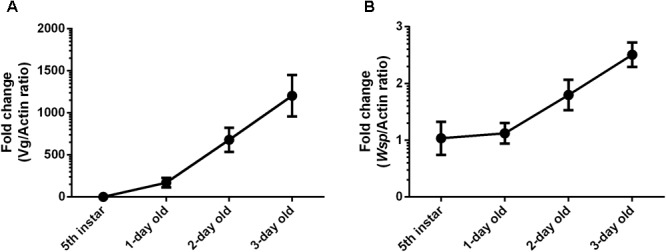
*Wolbachia* titers in *L. striatellus* ovaries at different developmental stages. **(A)** Vg gene transcript levels in *L. striatellus*. **(B)** *Wolbachia wsp* genome copies in *L. striatellus* ovaries. Vg mRNA expression levels in *L. striatellus* and *wsp* copy number in *L. striatellus* ovaries were measured in 5th instar nymphs and adults females at different emergence times (1, 2, and 3 days old). Results are expressed as fold change relative to 5th instar nymphs. Bars show the average fold change per experiment ± SD.

*Wolbachia* titers were detected in 5th instar nymphs and three stages of 1-, 2-, and 3-day-old adult female *L. striatellus* ovaries. The timing of Vg gene expression in *Wolbachia-*infected adult female *L. striatellus* was detected by RT-qPCR. *Wolbachia* titers in ovaries and Vg gene expression levels increased with female age at a background level in the 5th instar nymph (**Figure 2A**). Weakly increased *Wolbachia* titers were observed in the 1-day-old adult female when most ovarioles were in early vitellogenic stage. *Wolbachia* titers in ovaries and Vg expression sharply increased in the 2-day-old adult female and continuously increased in 3-day-old adult female. The higher Vg expression levels with age of female adults coincided with higher *Wolbachia* titers in the *L. striatellus* ovaries (**Figure 2B**), as expected based on previous results (Noda et al., 2001; Huo et al., 2014). These results suggest that higher *Wolbachia* titers in ovaries may be mediated by Vg transmission in *L. striatellus*.

### *Wolbachia* Are Transported as Ovaries Develop

To identify a possible functional role of Vg in the transovarial transmission of *Wolbachia*, we observed the presence of *Wolbachia* in ovaries and ovarioles at different developmental stages. We did not observe any *Wolbachia* signals in the ovarioles at the previtellogenic stage when Vg was not absorbed into ovaries. *Wolbachia* was found only in the terminal filaments and pedicels (**Supplementary Figure 1**). Ovaries in previtellogenic stage were difficult to keep intact during staining, and there was no whole ovaries image got.

**FIGURE 3:**
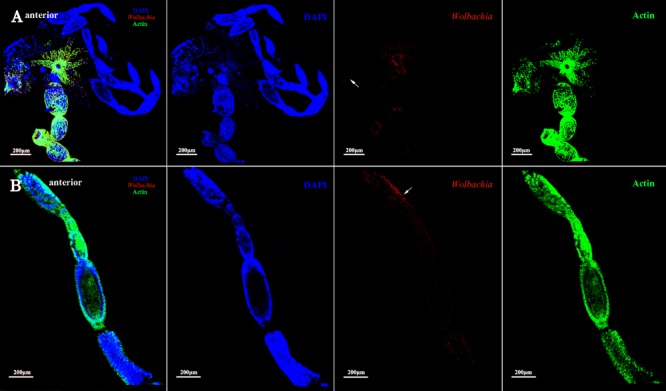
*Wolbachia* entering the early vitellogenic ovaries. Morphology of the structure and organization of ovaries **(A)** and ovarioles **(B)** of early vitellogenic adults stained with FITC-Phalloidin (green) and DAPI (blue). *Wolbachia* stained with an antibody to *Wolbachia* heat shock protein 60 (Hsp-60). Arrowheads point to *Wolbachia* aggregated within ovaries and ovarioles. Red: *Wolbachia*; green: actin; blue: *L. striatellus* DNA.

*Wolbachia* was first detected in the ovariole tropharium of adults at early vitellogenic ovaries when Vg began to be absorbed into ovaries (**Figure 3**). *Wolbachia* accumulated in the ovariole tropharium (**Figure 3B**, arrowhead) and was distributed among all cells (**Figure 3B**, arrowhead), including the centrally located trophic core and nurse cells (Szklarzewicz et al., 2007). These results suggest that *Wolbachia* may be transported into the tropharium at an early developmental stage, establishing an early infection.

**FIGURE 4:**
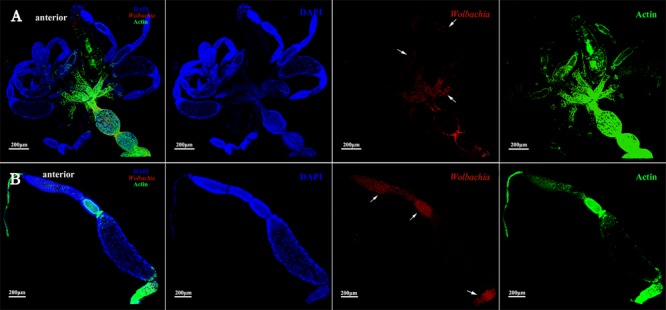
*Wolbachia* localization in vitellogenic I ovaries. *Wolbachia* in ovaries **(A)** and ovarioles **(B)** of vitellogenic I adults were observed by antibody Hsp-60 (red). White arrowheads point that *Wolbachia* within the posterior tropharium, anterior vitellarium, and pedicel. Red: *Wolbachia*; green: actin; blue: *L. striatellus* DNA.

The development of vitellogenic ovaries are divided into vitellogenic I and vitellogenic II stages according to ovarioles development. Vitellogenic I ovaries contain one developing oocyte. At this development stage, high quantities of Vg are incorporated into developing oocytes (Huo et al., 2014). *Wolbachia* signals were observed in the posterior tropharium, which is occupied by prefollicular cells and several arrested oocytes, and in the ooecium of anterior vitellarium, which had arrested and developing oocytes surrounded by follicular cells (**Figure 4**, arrowheads). These results show that *Wolbachia* migrated from the tropharium to the vitellarium in vitellogenic I ovarioles.

**FIGURE 5:**
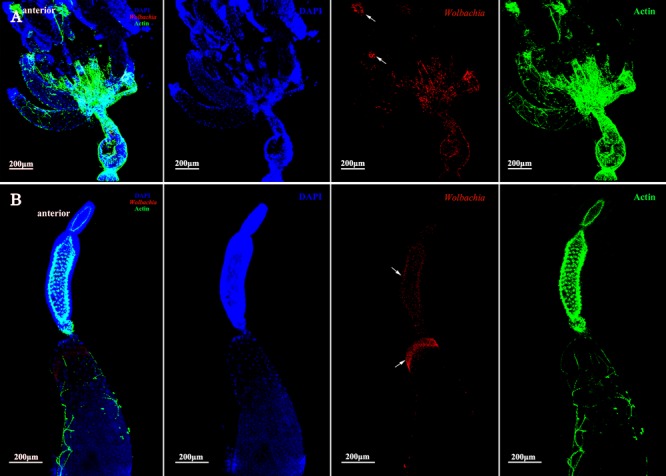
*Wolbachia* distribution in vitellogenic II ovaries. Ovaries **(A)** and ovarioles **(B)** of vitellogenic II adults were observed. White arrowheads indicate *Wolbachia* concentrated around the arrested oocytes and the anterior region of developing oocyte. Red: *Wolbachia*; green: actin; blue: *L. striatellus* DNA.

The vitellarium of vitellogenic II ovarioles house one developing oocyte which is nearly matured (**Figure 5A**). *Wolbachia* signals were concentrated at the anterior pole region in the developing oocytes (**Figure 5B**, arrowheads). *Wolbachia* were also observed in the ooecium of arrested oocytes. Obviously, *Wolbachia* signals concentrated around the arrested oocytes (**Figure 5B**, arrowheads), where is an area of nutritive cords connecting nurse cells. These observations suggest that *Wolbachia* has successfully invaded the developing oocytes from tropharium.

**FIGURE 6:**
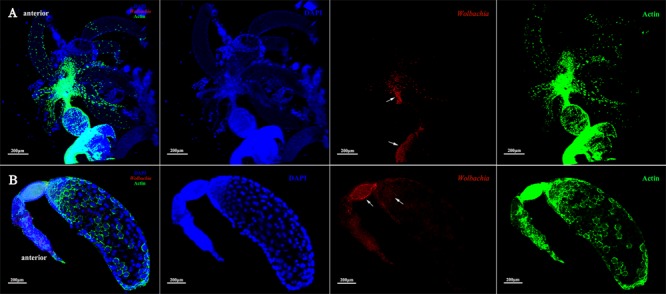
*Wolbachia* spread in postvitellogenic ovaries. Postvitellogenic ovaries **(A)** and ovarioles **(B)** infected with *Wolbachia* (red). Arrowheads point to *Wolbachia* localized in adult pedicels and oviducts. *Wolbachia* signals were also observed in the anterior vitellarium. Red: *Wolbachia*; green: actin; blue: *L. striatellus* DNA.

Ovarioles at postvitellogenic stage contain one matured oocyte and Vg transmission is nearly finished. At this developmental stage, most *Wolbachia* were observed in the anterior vitellarium (**Figure 6B**, arrowheads), in which the next oocytes were developing. *Wolbachia* in the matured oocytes kept the anterior region (**Figure 6B**, arrowheads). *Wolbachia* signals were also observed in the pedicel and oviduct (**Figure 6A**, arrowheads). These results suggest that *Wolbachia* transmission into oocytes have finished as oocytes matured.

### *Wolbachia* Transferred Into Oocytes From the Tropharium

**FIGURE 7:**
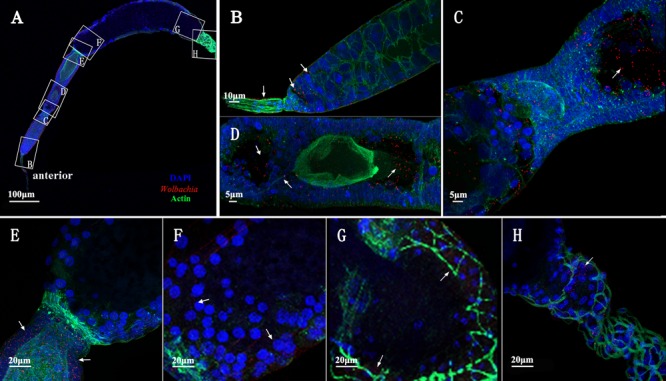
*Wolbachia* transferred into oocytes. **(A)** Representative ovariole include developing and matured oocytes. Higher magnification of the terminal filament and stem cells in the tropharium **(B)**, the connection between the tropharium and vitellarium **(C)**, developing oocyte **(D)**, the connection between developing and matured oocyte **(E)**, anterior region of the matured oocyte **(F)**, posterior region of the matured oocyte **(G)**, and pedicel **(H)**. White arrowheads indicate *Wolbachia*. Red: *Wolbachia*; green: actin; blue: *L. striatellus* DNA.

Immunohistochemistry using a *Wolbachia* Hsp-60 antibody revealed characteristic localization patterns of *Wolbachia* in the ovarioles, which were suggestive of the route and mechanism of its vertical transmission. At the tip of the ovarioles, the *Wolbachia* signals were found inside the terminal filament and nurse cells in the anterior pole of the tropharium (**Figures 7A,B**, arrowheads). We also show that *Wolbachia* are present in the extracellular space between nurse cells (**Figures 7A,B**, arrowheads). *Wolbachia* became dispersed throughout the ooecium of arrested oocytes (**Figures 7A,C**, arrowheads). However, a few arrested oocytes exhibited a residual accumulation of *Wolbachia* at the posterior region (**Figures 7A,D**, arrowheads), which may facilitate stable vertical transmission of *Wolbachia*. *Wolbachia* were also found in nutritive cords connected to nurse cells and developing oocytes (**Figure 7D**, arrowheads and **Supplementary Figure 2**), which indicated that *Wolbachia* in the nurse cells may spread into the developing oocytes binding with Vg protein through nutritive cords. The nutritive cords disappeared as the oocytes matured (**Figures 7A,E**, arrowheads). Many *Wolbachia* accumulated in the next developing oocytes rather than in matured oocytes (**Figures 7A,E,F**, arrowheads), which indicates that *Wolbachia* transmission was prevented during the completion of oocyte development. In matured oocytes, *Wolbachia* localized within the anterior region and the surrounding follicular cells (**Figures 7A,F,G**, arrowheads). Many *Wolbachia* were observed in the pedicels (**Figures 7A,H**, arrowheads), while smaller numbers were located in the posterior region of the matured oocytes (**Figures 7A,G**, arrowheads). These observations indicated that *Wolbachia* invaded oocytes from tropharium nurse cells on its anterior side rather than the pedicel side, pointing to a barrier between the pedicel and vitellarium preventing *Wolbachia* entry into the oocyte directly from the pedicel.

### Reduced Vg Expression Affected *Wolbachia* Invasion

**FIGURE 8:**
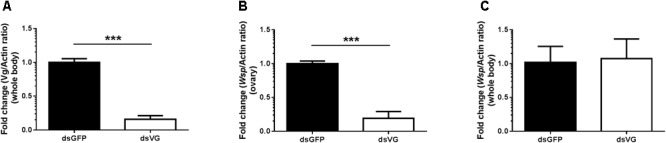
*Wolbachia* titers in dsVg-microinjected *L. striatellus*. Second instar nymphs were microinjected with Vg dsRNA or dsGFP as a control. After dsRNA injection, 2-day-old emerged female insects were selected to detect Vg expression in the whole body **(A)**. *Wolbachia* density significantly decreased in the dsVg-injected ovaries of 2-day-old adult insects **(B)** and did not differ in the whole body **(C)**. Bars show the average fold change per experiment ± SD. (^∗∗∗^*P* < 0.001).

To confirm that Vg has a role in transovarial transmission of *Wolbachia*, we knocked down Vg expression using RNAi by microinjecting dsRNA for Vg (dsVg) into the *L. striatellus* body with dsGFP as a control. Compared with dsGFP-microinjected insects, the Vg mRNA level in dsVg-microinjected 2-day-old emerged female insects decreased by approximately 87% throughout the whole body (**Figures 8A**). We used *Wolbachia wsp* qPCR primers to measure the total number of *wsp* copies, which revealed that the *Wolbachia* titers significantly decreased by 80% in the dsVg-microinjected insect ovaries compared with that of the control (**Figure 8B**). However, the *Wolbachia* titers in the whole body of dsVg-microinjected insects did not differ greatly among the treatments (**Figure 8C**), which indicates that knockdown of Vg expression by RNAi specifically affected *Wolbachia* invasion in the *L. striatellus* ovaries. Moreover, the dsVg-treated females failed to produce offspring (**Supplementary Figure 3**), consisting with previous finding (Huo et al., 2014).

**FIGURE 9:**
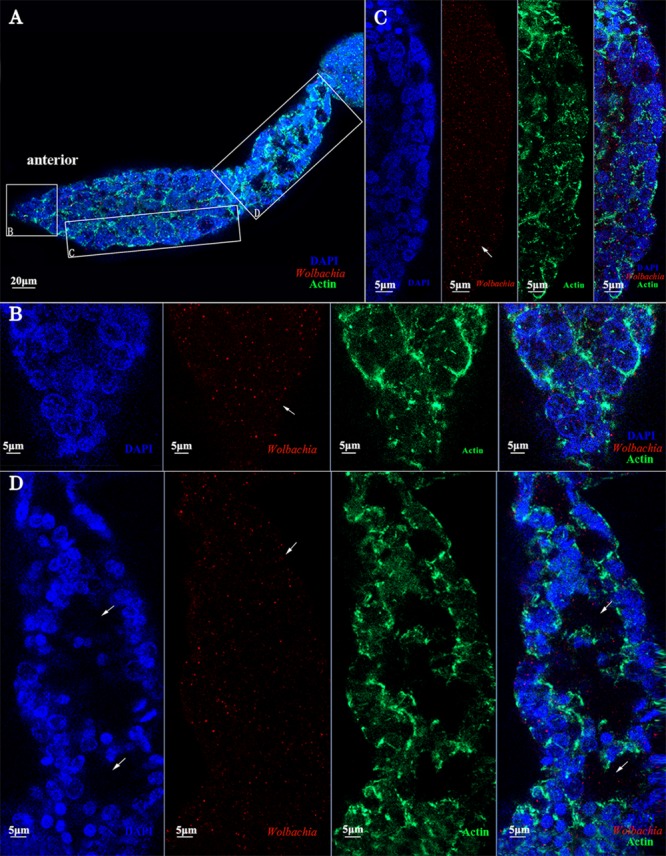
dsVg microinjection reduced the *Wolbachia* titer in ovaries. Low *Wolbachia* densities were detected in ovarioles of dsVg-treated 2-day-old emerged female insects **(A)**, including in the terminal filament **(B)**, tropharium **(C)**, and vitellarium **(D)**. *Wolbachia* dispersed throughout the whole ovariole irregularly. Red: *Wolbachia*; green: actin; blue: *L. striatellus* DNA.

We did not observe differences in degree of development among ovaries dissected from the treated insects. However, as Vg expression was reduced after dsVg injection, the ooecium of oocyte was smaller, and the oocyte in each ovariole was less distinct than that in the dsGFP-treated insects (**Figures 9A,D**, arrowheads and **Supplementary Figure 4**). *Wolbachia* distribution in the 2-day-old treated adult insect ovarioles was examined. Using immunofluorescence, we observed that *Wolbachia* were irregularly dispersed throughout the whole ovariole, including in the terminal filament (**Figures 9A,B**, arrowheads), tropharium (**Figures 9A,C**, arrowheads), and vitellarium (**Figures 9A,D**, arrowheads). The *Wolbachia* densities were lower in dsVg-treated than in the dsGFP-treated ovarioles (**Supplementary Figure 5**). All of these results suggest that Vg expression reduction by RNAi prevented *Wolbachia* transmission into *L. striatellus* ovaries.

## Discussion

*Wolbachia* infect an estimated 40–69% of arthropod species (Hilgenboecker et al., 2008). For *Wolbachia* to become established in a new host species, it must be horizontally acquired and vertically transmitted at a high rate (Turelli and Hoffmann, 1995). Regardless of how *Wolbachia* reach a new host, after the initial infection, they must reach the germ line for successful transmission to the next generation (Werren et al., 2008). Hence, their presence in the oocyte is essential for their vertical propagation to the next generation. In *Drosophila, Wolbachia* entry into oocyte has been intensively investigated (Ferree et al., 2005; Newton et al., 2015). During early oogenesis, *Wolbachia* are distributed among all of the germ cells in the germarium of ovarioles, allowing them to establish infection very early in development (Ferree et al., 2005). Within the germarium, the major route for *Wolbachia* to enter the germ cells is through the somatic stem cell niche. While little is known about these mechanisms and no mutation blocking *Wolbachia* vertical transmission has been previously described.

In the present study, we have investigated transmission in *L. striatellus*, which efficiently transmits *Wolbachia* given that all individuals tested so far were infected with *Wolbachia w*Stri (Noda, 1984; Zhang et al., 2013). In this study, a high correlation was found between Vg and *Wolbachia*. *Wolbachia* may bind with Vg in the hemolymph and then be absorbed into ovaries via Vg receptor-mediated endocytosis. Before Vg were absorbed into ovarioles, *Wolbachia* was only observed in the terminal filaments and pedicels of previtellogenic ovaries (**Supplementary Figure 1**). As ovaries development, *Wolbachia* were dispersed throughout all cells in the tropharium of early vitellogenic ovaries (**Figure 3**) when Vg were absorbed into the ovaries from hemolymph via endocytosis. Then *Wolbachia* spread into all ooecium at vitellogenic and postvitellogenic stages (**Figures 4–6**). Moreover, the density of *Wolbachia* in the ovarioles increased with development (**Supplementary Figure 6**). It was obvious that *Wolbachia* location in ovarioles was correlated with the transportation of Vg, which suggested that Vg is important for *Wolbachia* to overcome the transovarial transmission barrier.

In insect, there is a transovarial transportation system for absorbing nutrients Vg (Huo et al., 2018). *Wolbachia* in *L. striatellus* may use Vg existing transport systems for efficiently transovarial transmission. *Wolbachia* and Vg bind together in the hemolymph, and were transported into the tropharium via wide channels formed between nurse cells, endocytosed into the central trophic core establishing an early infection at an early developmental stage. The distribution of *Wolbachia* in the ovarioles of dsVg-injected *L. striatellus* supports our results. Compared with the dsGFP-microinjected insects, dsVg injection obviously affected *Wolbachia* invasion into the ovaries (**Figure 9**). *Wolbachia* were irregularly dispersed throughout the whole ovariole (**Figure 9**), and the *Wolbachia* titer in the ovarioles was greatly decreased (**Supplementary Figure 5**). These results further suggested that Vg was specifically involved in *Wolbachia* invasion of the insect ovaries. However, the detailed functions of Vg in *Wolbachia* transmission and how *Wolbachia* binds to Vg remain unclear.

We first observed *Wolbachia* signals in the ovarioles of adults at early vitellogenic ovaries when Vg began to be transferred into ovaries (**Figure 3**). At this stage, *Wolbachia* was distributed among all cells in the anterior area of tropharium, including the centrally located trophic core and nurse cells (**Figure 3B**, arrowhead). In fruit fly, the anterior area of ovarioles is called germarium, where contain stem cells and further differentiate into oocytes and nurse cells (Ferree et al., 2005). *Wolbachia* reach oocytes through the stem cells in the germarium (Toomey et al., 2013). While oocytes are mainly differentiated from stem cells in *L. striatellus* nymph stage, when Vg is not highly expressed (Huo et al., 2014). Therefore, despite *Wolbachia* invading the tropharium at early vitellogenic stage *L. striatellus*, it does not seem possible that *Wolbachia* spreads to the oocytes from the stem cells as *Wolbachia* does in *D. melanogaster*.

*Wolbachia* exhibited movement within nurse cells of *D. melanogaster* (Ferree et al., 2005). In this study, *Wolbachia* was also observed in nurse cells of *L. striatellus* ovarioles. Nurse cells in telotrophic meroistic ovarioles usually provide nutrients to the developing oocytes via nutritive cords. Vg was found in the nurse cells of *L. striatellus* by electron and immunoelectron microscopy (Huo et al., 2014). *Wolbachia* and Vg may be transported into the nurse cells together via endocytosis. In the nurse cells, Vg was processed into yolk granules (Rouillé et al., 1995) and *Wolbachia* was released into the cytoplasm. Finally, *Wolbachia* in the nurse cells spread into the developing oocytes as Vg flows through the nutritive cords. This scenario could explain why *Wolbachia* accumulation in nutritive cords (**Figure 7D** and **Supplementary Figure 2**).

Endosymbiotic organisms in *L. striatellus* appear to use different pathways for transovarial transmission. YLSs pass through the ovariole pedicel and enter the oocyte through a deep depression at the posterior pole (Noda, 1974, 1977). RSV does not directly invade the oocyte from the terminal filament or the pedicel, but instead invades nurse cells at the tip of the tropharium and then spreads into oocytes as the ovaries development (Huo et al., 2014). Bacteriocytes (*Nysius ericae, Nithecus jacobaeaein, Marchalina hellenica*, and *Scaphoideus titanus*) in insects with telotrophic ovarioles enter the tropharium and then infect oocytes through the nutritive cords (Sacchi et al., 2008; Swiatoniowska et al., 2013; Szklarzewicz et al., 2013). Tightly packed endosymbiotic microorganisms were observed in the apex tropharium of *Cixius nervosus* (Szklarzewicz et al., 2007). Actin in the polytrophic ovarioles may affect the location of *Wolbachia* in the *Drosophila* oocytes (Ferree et al., 2005; Newton et al., 2015). In *D. melanogaster, Wolbachia* enter into ovaries from the anterior tip of the germarium, which includes a terminal filament and cap cells (Frydman et al., 2006). The *Wolbachia* pattern of strong tropism in host ovaries was controlled by evolutionarily conserved *Wolbachia*-encoded factors (Toomey et al., 2013). In *L. striatellus, Wolbachia* were first found in the anterior tropharium of ovarioles (**Figure 3**). *Wolbachia* invaded each ovariole from tropharium on its anterior side rather than the pedicel side (**Figure 7**), pointing to a barrier between the pedicel and vitellarium preventing *Wolbachia* entry into the oocyte directly from the pedicel.

**FIGURE 10:**
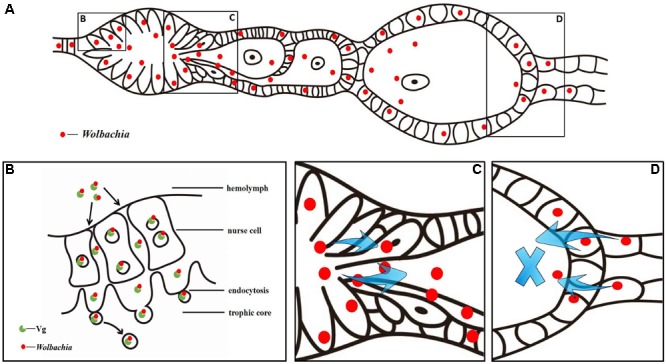
*Wolbachia* transovarial transmission model. **(A)** Diagram of *Wolbachia* concentrated in an *L. striatellus* ovariole. *Wolbachia* invade the ovariole from tropharium cells of its anterior side **(B)** via wide channels formed between nurse cells and migrate from the tropharium to the oocytes through the nutritive cord **(C)**. *Wolbachia* invaded the ovariole from its anterior side rather than the pedicel side **(D)**.

Based on the above observations, we propose a model for *Wolbachia* transovarial transmission into *L. striatellus* oocytes (**Figure 10**). *Wolbachia* bind to Vg outside the ovarioles and then endocytosed into nurse cells and central located trophic core in the tropharium via wide channels formed between nurse cells during the early phase of vitellogenesis (**Figures 10A,B**). As central located trophic core divide into oocytes, some *Wolbachia* enter the arrested oocyte and establish an early infection. *Wolbachia* in the nurse cells spread into the developing oocytes through the nutritive cords with or without binding with Vg (**Figures 10A,C**). However, *Wolbachia* transmission is prevented when the oocyte is completely developed and the nutritive cords disappear. This model suggests that *Wolbachia* invade the *L. striatellus* ovariole from anterior tropharium cells rather than the pedicel side (**Figures 10A,D**), and use the existing Vg transovarial transport system to enter into oocytes for efficient maternal transmission.

Due to lack of anti-Vg monoclonal and polyclonal antibody in *L. striatellus*, RT-qPCR, qPCR, RNAi and immunofluorescence staining were used to point to interactions between the *Wolbachia* infection and the presence of host Vg. These interactions may play a role in *Wolbachia* motility and replication in the host, and thus may be responsible for the efficient maternal transmission of *Wolbachia*. Further studies to characterize *Wolbachia* proteins that bind to or alter host Vg are needed to understand the molecular basis of the interaction between *Wolbachia* and egg development.

## Author Contributions

YG, AH and X-YH wrote the manuscript. X-QX, P-WM, and H-JH performed the experiment. J-TG and J-FJ performed the images processing.

## Conflict of Interest Statement

The authors declare that the research was conducted in the absence of any commercial or financial relationships that could be construed as a potential conflict of interest.
