# Supplementary material for: Vertical Transmission of Wolbachia Is Associated With Host Vitellogenin in Laodelphax striatellus
Source: Front Microbiol. 2018 Aug 28;9:2016. doi: 10.3389/fmicb.2018.02016 (PMC6127624; doi:10.3389/fmicb.2018.02016)
Supplement: Supplementary file 1 [file Data_Sheet_1.PDF]

Supplementary Material

Vertical Transmission of *Wolbachia* is Associated with Host Vitellogenin in *Laodelphax striatellus*

Yan Guo[1], Ary A. Hoffmann[2], Xiaoqin Xu[1], Peiwen Mo[1], Haijian Huang[1], Juntao Gong[1], Jiafei Ju[1], and Xiaoyue Hong[1*]

[*] Correspondence:

Dr. Xiaoyue Hong, Department of Entomology, Nanjing Agricultural University, Nanjing, Jiangsu, China, xyhong@njau.edu.cn

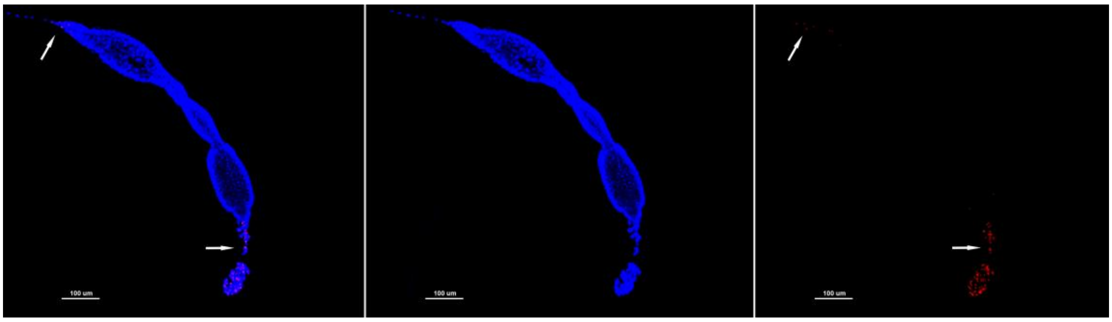

Supplementary Figure 1. *Wolbachia* in the previtellogenic ovarioles. Arrowheads point to *Wolbachia* localized in the terminal filament and pedicel in previtellogenic ovarioles. Actin did not be scanned due to its packed tightly at this stage. Red: *Wolbachia*; blue: *L. striatellus* DNA.

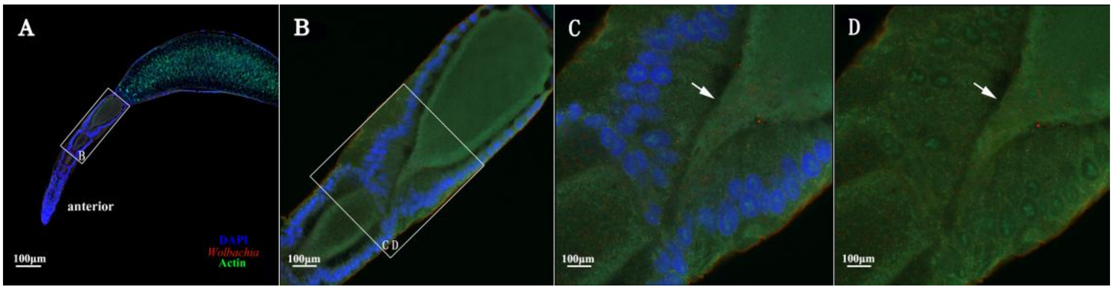

Supplementary Figure 2. *Wolbachia* in the nutritive cord of *L. striatellus*. *Wolbachia* migrate with trophic flow from the tropharium to the oocytes through the nutritive cord. White arrowheads indicate *Wolbachia* in the nutritive cord. Red: *Wolbachia*; green: actin; blue: *L. striatellus* DNA.

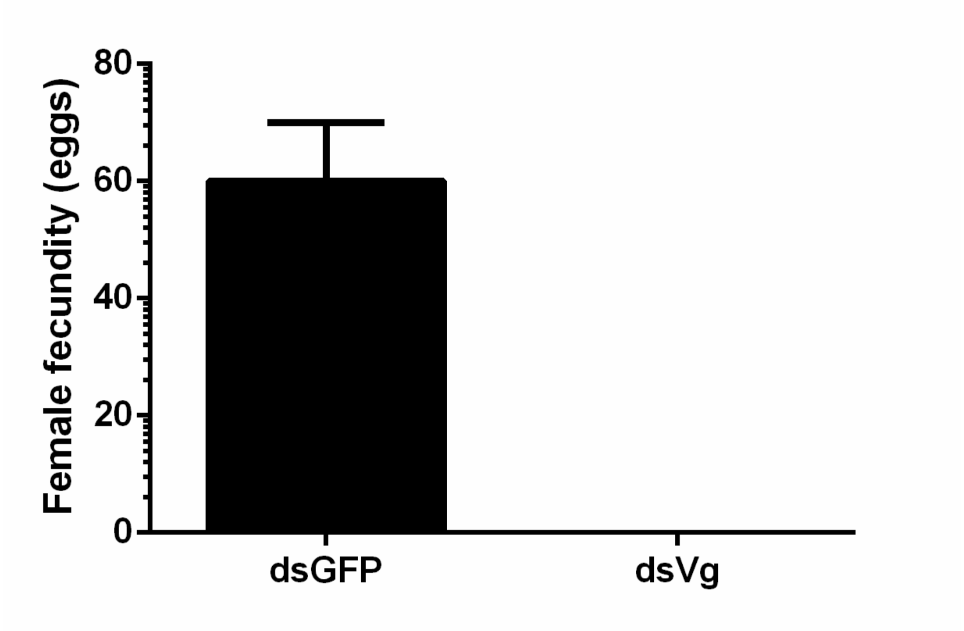

Supplementary Figure 3. Impact of dsVg on *L. striatellus* fecundity. The reproduction of dsVg *L. striatellus* was examined. 20 newly emerged dsVg and dsGFP *L. striatellus* were randomly selected. Each pair was introduced into a glass beaker (7-cm diameter × 14-cm height) with rice seedlings. Seedlings were changed every 24 h, and eggs were counted by gently stripping the sheaths under a binocular stereomicroscope. Eggs were counted until the female died, and newly emerged males were supplemented if the original male died before the experiment ended; bar show the average eggs per female ± SD.

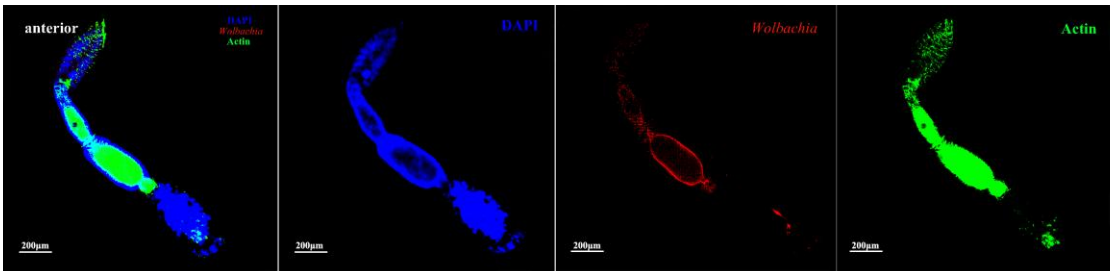

Supplementary Figure 4. *Wolbachia* in dsGFP-treated ovarioles. *Wolbachia* distribution in the 2-day-old dsGFP-treated adult insect ovarioles was examined. Red: *Wolbachia*; blue: *L. striatellus* DNA.

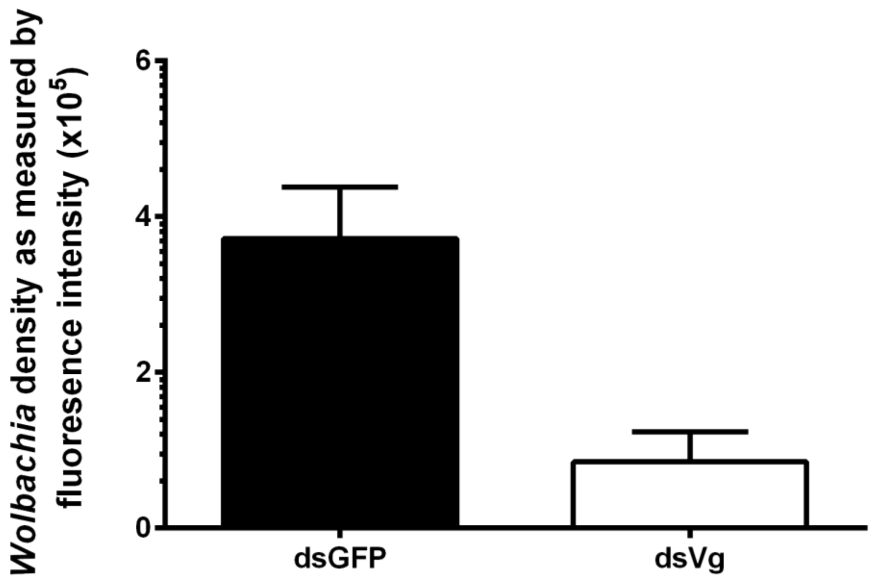

Supplementary Figure 5. Impact of dsVg on *Wolbachia* densities in ovarioles. Second instar nymphs *L. striatellus* microinjected with Vg dsRNA had emerged for 48h, which were selected to detect *Wolbachia* density in ovarioles. *Wolbachia* densities were measured by fluorescence intensity; average of six ovarioles ±SD.

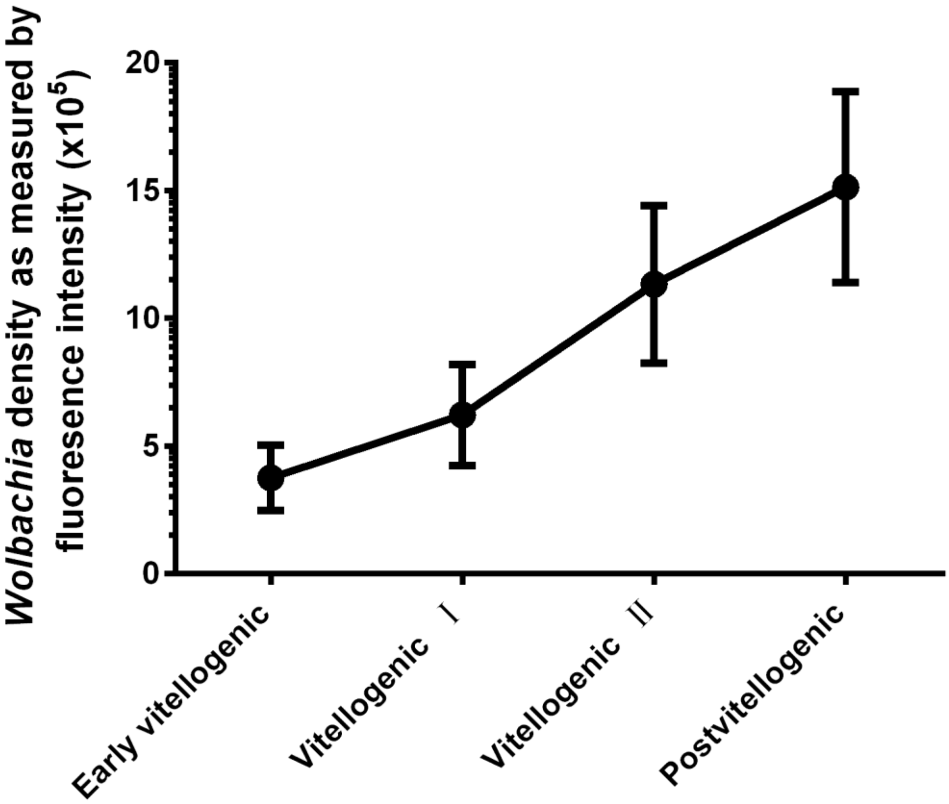

Supplementary Figure 6. *Wolbachia* titers in ovarioles of *L. striatellus* at different development stages. *Wolbachia* density in early vitellogenic, vitellogenic Ⅰ, vitellogenic Ⅱ, and postvitellogenic stages were measured by fluorescence intensity; average of six ovarioles ±SD.

Supplementary Table 1. Primers used in quantitative real-time PCR, quantitative PCR, and dsRNA.

**Genes**	**Sequence (5′-3′)**
**Primers used in quantitative real-time PCR**	
**Vg-sense**	ACCTTGTCAACCGTGCCTCA
**Vg-antisense**	GCCAACTACTCCCTTCTGCTT
**Actin-sense**	CGCGATCTGACCGACTACCT
**Actin-antisense**	GTAGCACAGTTTCACCTTGATGTCT
**Primers used in quantitative PCR**	
***wsp*-sense**	GTTGATGTTGAAGGGCTTTACTCAC
***wsp*-antisense**	GGCATATCTTCAATCGCTATATCGT
**Actin-sense**	CGCGATCTGACCGACTACCT
**Actin-antisense**	GTAGCACAGTTTCACCTTGATGTCT
**Primers used in double stranded RNA synthesis**	
**dsVg-sense**	TAATACGACTCACTATAGGGCACTGGTCATCTTCCACCCT
**dsVg-antisense**	TAATACGACTCACTATAGGGGAGTAGGTACCGCAGAGACC
**dsGFP-sense**	TAATACGACTCACTATAGGGAGAATGAGTAAAGGAGAAGAACTTTTC
**dsGFP-antisense**	TAATACGACTCACTATAGGGAGATTTGTATAGTTCATCCATGCCATGT
